# Draft Genome Sequence of *Lactobacillus brevis* Strain EW, a *Drosophila* Gut Pathobiont

**DOI:** 10.1128/genomeA.00938-13

**Published:** 2013-11-21

**Authors:** Eun-Kyoung Kim, Young Min Park, Oun Young Lee, Won-Jae Lee

**Affiliations:** School of Biological Science, Seoul National University and National Creative Research Initiative Center for Symbiosystem, Seoul, South Korea

## Abstract

*Lactobacillus brevis* strain EW, a gut pathobiont, was isolated from the fruit fly *Drosophila melanogaster*. Here, we report the draft genome sequence of *L. brevis* EW.

## GENOME ANNOUNCEMENT

*Lactobacillus brevis* is a Gram-positive rod-shaped species of lactic acid-producing bacteria. Many strains of *L. brevis*, such as ATCC 367 (1), KB290 (2), and *L. brevis* subsp. *gravesensis* ATCC 27305, have been shown to exert a probiotic effect in humans and animals. *L. brevis* strain EW was originally isolated from the gut epithelia of *Drosophila melanogaster* (3). Although the presence of lactobacilli is generally considered to be beneficial to the host, *L. brevis* EW acts as a pathobiont (i.e., a resident gut bacterium that is generally benign within a normal community structure but is pathogenic under certain deregulated conditions) in the *Drosophila* gut (4). A recent study showed that germfree *Drosophila* monoassociated with *L. brevis* EW exhibited severe gut cell apoptosis and early host death (4). The *L. brevis* EW-induced host pathology is a result of the chronic activation of a reactive oxygen species-producing enzyme, dual oxidase (DUOX). Since bacterium-derived uracil acts as a ligand for DUOX activation in *Drosophila* gut immunity (4), it has been hypothesized that *L. brevis* EW releases uracil in a constitutive manner, which in turn induces chronic DUOX-dependent reactive oxygen species (ROS) generation in the gut. However, it is unclear as to why *L. brevis* EW secretes uracil, as certain other lactobacilli do not. In the current study, the genomic information for *L. brevis* EW is presented, which is essential for elucidating the mechanism by which *L. brevis* EW provokes gut-microbe dysbiosis through chronic DUOX activation.

The genome sequence of *L. brevis* EW was determined using a 3-kb paired-end library (175,405 reads, ~10.92-fold coverage) with the Genome Sequencer FLX system (Roche Diagnostics, Branford, CT) and a 100-bp paired-end library (6,924,211 reads, ~362.4-fold coverage) with the Illumina HiSeq 2000 (Illumina, San Diego, CA). The 38 contigs were generated using the CLC Genomics Workbench 5.1 (CLC bio, Denmark). The functional annotation of the predicted genes was performed using the RAST server (5) and the COG database (6). The draft genome sequence is 2,885,101 bp in length and contains 2,892 open reading frames (ORFs). Eleven rRNA genes and 68 tRNA genes were also identified. The G+C content of the genome is 45.26 mol%. Using comparative genomics for the various *L. brevis* strains with different characteristics, i.e., regarding their roles as symbionts or pathobionts within the *Drosophila* gut, it is now feasible to dissect the molecular principles governing gut-microbe symbiosis and pathogenesis.

### Nucleotide sequence accession numbers.

The result of this whole-genome shotgun project has been deposited at DDBJ/EMBL/GenBank under the accession no. AUTD00000000. The version described in this paper is the first version, AUTD01000000.
